# Meralgia paraesthetica

**DOI:** 10.1016/j.bjae.2025.04.006

**Published:** 2025-06-07

**Authors:** K. Bromley, S. Kanakarajan

**Affiliations:** Aberdeen Royal Infirmary, Aberdeen, UK

**Keywords:** chronic pain, femoral neuropathy, meralgia paraesthetica, pain, pain management

**Learning objectives**

By reading this article you should be able to:

• Explain the clinical presentation of meralgia paraesthetica (MP) and its impact on patients.
• Describe the incidence, prevalence and risk factors for MP.
• Detail the anatomical variations of the lateral femoral cutaneous nerve (LFCN).
• Illustrate the diagnostic tools and various treatment options for managing MP.

**Key points**

• MP is a condition causing pain and paraesthesia in the anterolateral thigh.
• The incidence of MP is 4.2–32.6 per 100,000 patient years, with higher risks in males, pregnant women and those living with obesity or diabetes.
• The variable anatomical path of the LFCN influences the presentation and diagnosis of MP.
• MP is diagnosed through clinical symptoms, supported by imaging and electrophysiological studies, with ultrasound being particularly effective.
• Most patients can be managed conservatively but a small proportion may benefit from interventional procedures including injections, electrical stimulation and surgery.

Meralgia paraesthetica (MP) is a common but under-recognised mononeuropathy of the lateral femoral cutaneous nerve (LFCN), leading to dysaesthesia in the anterolateral distribution of the thigh. Patients with MP experience paraesthesia and pain with or without loss of sensation. A lack of knowledge and delay in diagnosing MP can significantly disable patients and impair their quality of life.^1^

In 1885, Hager described an association between the compression of the LFCN and pain.^2^ In 1895, both Bernhardt and Roth published more comprehensively on the association. For some time, it was known as Bernhardt–Roth syndrome, but later Roth coined the term meralgia paraesthetica. MP is a term derived from the Greek words *meros* (thigh) and *algos* (pain).^3^^,^^4^ This article aims to provide an overview of the epidemiology, related anatomy, aetiology, diagnosis and treatment of MP.

## Epidemiology

A population-based study by Parisi and colleagues in the USA between 1990 and 1999 found the incidence of MP to be 32.6 per 100,000 patient years.^5^ A similar study carried out in the Netherlands reported an incidence of 4.3 per 100,000 patient years.^6^ Parisi and colleagues found an association between diabetes mellitus and MP. Moreover, they found the incidence of MP to be 7.5 times higher amongst patients with diabetes mellitus compared with the general population. The authors also reported that patients with MP were twice as likely to have diabetes mellitus than control subjects matched for age, sex and BMI. The mean BMI in patients with MP was significantly higher at 30.1 kg m^−2^ compared with 27.3 kg m^−2^ for the normal population, postulating a link between MP and obesity.^5^ van Slobbe and colleagues found MP to be associated with both pregnancy and carpal tunnel syndrome.^6^ Moreover, 14.5% of patients in their study had previously been pregnant. Meralgia paraesthetica was also found to be most common in those aged 41–60 yrs with a slight male preponderance.^6^

## Aetiology

The causes of MP can be classified as idiopathic or iatrogenic (Table 1). Idiopathic causes can be mechanical or metabolic. Direct entrapment of the LFCN is the most common mechanical cause.^7^ Other mechanical factors relating to anatomy include leg length discrepancy and degenerative disease of the pubic symphysis. Pregnancy, obesity, retroperitoneal tumours, abdominopelvic tumours and abdominal fluid may lead to MP because of increased intra-abdominal pressure. In addition, belts, corsets and tight trousers have also been implicated as mechanical causative factors because of external pressure. Lead poisoning, diabetes mellitus and alcoholism are metabolic factors that cause inflammation and subsequent damage to the LFCN.^8^^,^^9^

**Table 1:** Causes of meralgia paraesthetica.

Cause	Type/examples
Idiopathic	Mechanical
	• Abdominal distension from ascites or tumours
	• Belts and corsets
	• Leg length discrepancy
	• Obesity
	• Pregnancy
	• Tight clothing
Metabolic	
	• Alcoholism
	• Diabetes mellitus
	• Lead poisoning
Iatrogenic	Indirect trauma
	• Gynaecology—oncology surgeries
	• Lower segment Caesarean section
	• Prolonged prone positioning (e.g. in ICU)
	• Spinal surgeries requiring prone positioning
Direct trauma	
	• Intramuscular injections
	• Inguinal hernia repair

Meralgia paraesthetica has been associated with many surgical procedures. Although gynaecology oncological surgery is associated with MP, it is unlikely that this is a result of direct trauma as the LFCN runs outside the surgical field. However, the use of self-retaining retractors, nerve compression in the lithotomy position and the formation of a psoas muscle haematoma can contribute to the evolution of MP.^10^ Similarly, lower segment Caesarean section has been implicated in causing MP as a result of prepositioning in the lithotomy position for attempted vaginal delivery and more rarely, i.m. injection of drugs into the thigh.^11^^,^^12^ The use of either the Jackson spinal surgery table or the Relton–Hall frame for prone positioning in posterior spinal surgery has been associated with the development of MP. Both operating tables cause external compression, reducing blood flow in the vasa nervorum of the LFCN leading to subsequent nerve dysfunction. Although the supportive structures are padded, additional protection using egg-crate foam and gel pads should be used when appropriate and vigilance should be applied when positioning the angle of the patient's hips.^13^ The use of prone positioning in the intensive care unit to optimise ventilation has been associated with MP.^14^ The LCFN can be damaged in many surgical procedures including pelvic osteotomy, laparoscopic cholecystectomy, laparoscopic myomectomy, coronary artery bypass grafting, gastric reduction surgery and inguinal hernia repair.^9^

## Anatomy

The LFCN is a sensory nerve arising from the dorsal branches of the lumbar ventral rami. The LFCN most commonly comprises the L2 and L3 nerve roots (58.5%) but cadaveric studies have shown that it can also arise from L1 and L2 or from L2 alone.^15^^,^^16^ It is important to consider the variation in the origin of the LFCN and thus, the potential for varying clinical presentation so that MP is not prematurely disregarded as a potential diagnosis.

**Fig 1:**
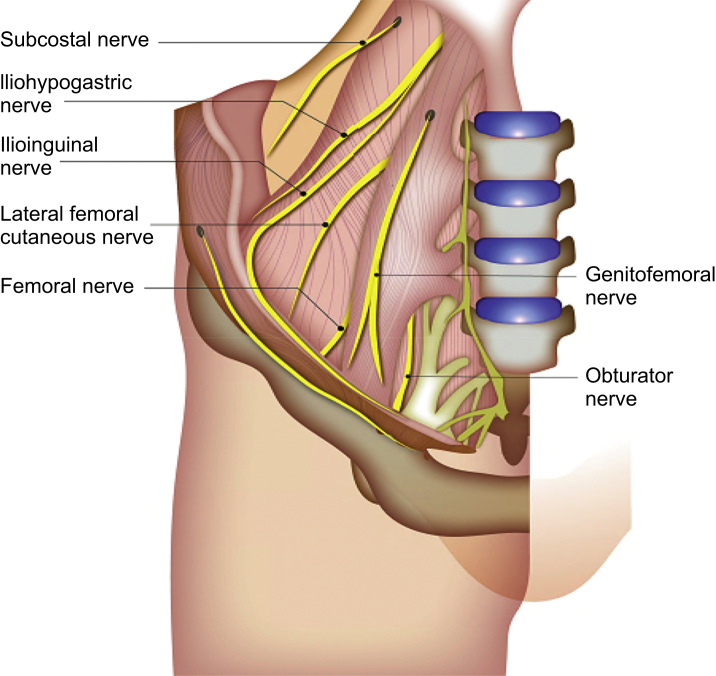
The course of the lateral femoral cutaneous nerve.

**Fig 2:**
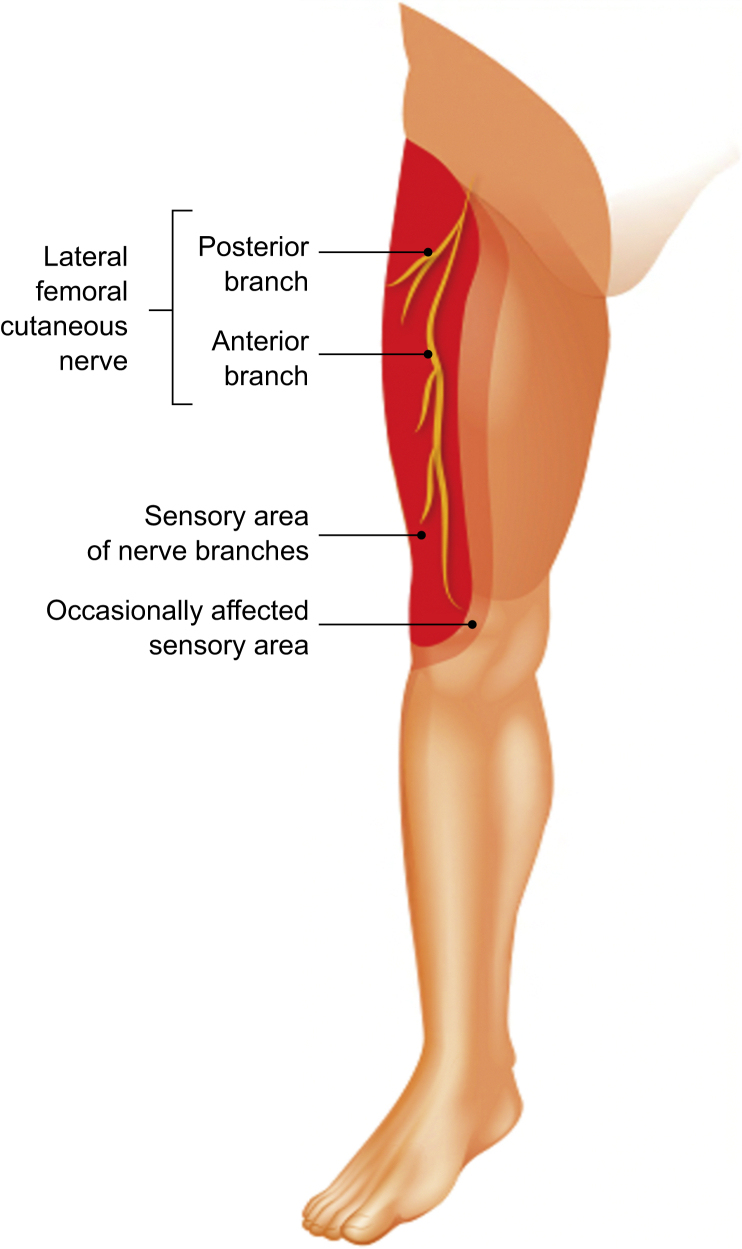
The branches of the lateral femoral cutaneous nerve and area of sensory innervation.

The LFCN emerges near the lateral border of the psoas major muscle at (30%), above (27%), or below (43%) the level of the iliac crest.^17^ It then runs between the iliac fascia and iliacus towards the anterior superior iliac spine (Fig. 1). In its course, the LFCN may pass under, through or above the inguinal ligament to enter the anterior thigh.^7^ The distance between the anterior superior iliac spine and the inguinal ligament at which the LFCN passes is variable, with a mean distance of 1.73 cm.^18^ Distal to the inguinal ligament, the LFCN divides into the anterior and posterior branches to supply the anterolateral thigh (Fig. 2).^7^

The course of the LFCN varies greatly after it leaves the pelvis. Cadaver studies enabled Aszmann and colleagues to construct a classification system for the five anatomical routes of the LFCN (Table 2).^19^ They postulated that LFCN damage was more common in types A to C where the nerve is superficial to the inguinal ligament and more vulnerable to direct mechanical trauma and surgery.^19^ However, ultrasound studies have since implicated type B as the most common anatomical variant amongst patients with MP.^20^

**Table 2:** Classification system used to describe the route of the lateral femoral cutaneous nerve.^19^

Class	Percentage	Anatomical route
Type A	4	Posterior to the anterior superior iliac spine, across the iliac crest
Type B	27	Anterior to the anterior superior iliac spine and superficial to the origin of the sartorius muscle but, within the substance of the inguinal ligament
Type C	23	Medial to the anterior superior iliac spine, ensheathed in the tendinous origin of the sartorius muscle
Type D	26	Medial to the origin of the sartorius muscle located in an interval between the tendon of the sartorius muscle and thick fascia of the iliopsoas muscle deep to the inguinal ligament
Type E	20	Most medial and embedded in loose connective tissue, deep to the inguinal ligament, overlying the thin fascia of the iliopsoas muscle, and contributing to the femoral branch of the genitofemoral nerve

## Diagnosis

Meralgia paraesthetica can be diagnosed on clinical grounds in patients presenting with numbness, tingling, pain, burning and dysaesthesia over the anterolateral aspect of the thigh.^7^ Patients often report that their symptoms may be exacerbated by standing, walking or extension of the leg at the hip. Presentation is usually unilateral but can be bilateral in 20% of cases.^21^

Neurological examination in patients with MP will reveal sensory dysfunction confined mostly to the anterolateral thigh. However, the wide anatomical variation in the nerve root supply to the LFCN may mean that there is some crossover in sensory disturbance to the medial aspect of the leg and it is important to not dismiss MP as a potential diagnosis if the patient does not present with the classical pattern of sensory disturbance. Any motor loss or altered reflexes should prompt consideration of an alternative diagnosis.^7^ The differential diagnosis of MP includes femoral nerve neuropathy, lumbar plexopathy and lumbar radiculopathy. Needle electrode examination, ultrasound scan, X-ray, CT scan and MRI can be used to exclude potential differential diagnosis.^7^

Electrophysiological studies may be useful when there is uncertainty regarding the diagnosis. Although such studies are commonly used to confirm the diagnosis of MP for interventional studies, they are seldom used in clinical practice. The uses of the sensory nerve action potential (SNAP), sensory nerve conduction velocity (SNCV) and somatosensory evoked potentials (SSEPs) have been described in the literature.^22^

Ultrasound confers many advantages over electrophysiological studies in the diagnosis of MP. Ultrasound can identify morphological nerve changes and identify anatomical variations of the LFCN. A retrospective population-based study investigating the sonographic features of the LFCN in MP found that the LFCN may appear hypoechogenic with abnormal intraneural vascularity. In addition, an abrupt change in the calibre of the nerve may occur with an increase in cross-sectional area. The average cross-sectional area of the LFCN was found to be 1.70 mm^2^ and the authors considered a cross-sectional area of 2.65 mm^2^ to be pathological.^23^ Finally, ultrasound-guided infiltration with local anaesthetic (LA) around the LFCN can help to diagnose MP as symptoms subside after infiltration.

## Treatment

### Conservative

Meralgia paraesthetica is often self-limiting with complete resolution in 91% of patients managed conservatively. Reassurance should be provided when symptoms are mild.^21^ Persistent symptoms should be managed conservatively by avoiding tight-fitting clothing, physiotherapy, weight loss in those with obesity, medication and ultrasound-guided infiltration with LA and corticosteroid.^24^ Medication treatment options include simple analgesics, antineuropathic medications, tricyclic antidepressants and neuromuscular blocking drugs.^25^

### Ultrasound-guided infiltration

Infiltration of the LFCN can be carried out using a landmark technique or under ultrasound guidance. The former has been associated with a failure rate of 60% because of the vast anatomical variation of the course of the LFCN and hence, the lack of a predictable relationship with surface landmarks.^26^ A study in 20 cadavers and 10 healthy volunteers showed that the anatomical landmark technique had a location accuracy of 5.3% in cadavers and 0% in healthy volunteers, but ultrasound location accuracy was 84.2% in cadavers and 80% in healthy volunteers, confirming the superiority of the use of ultrasound guidance.^27^

**Fig 3:**
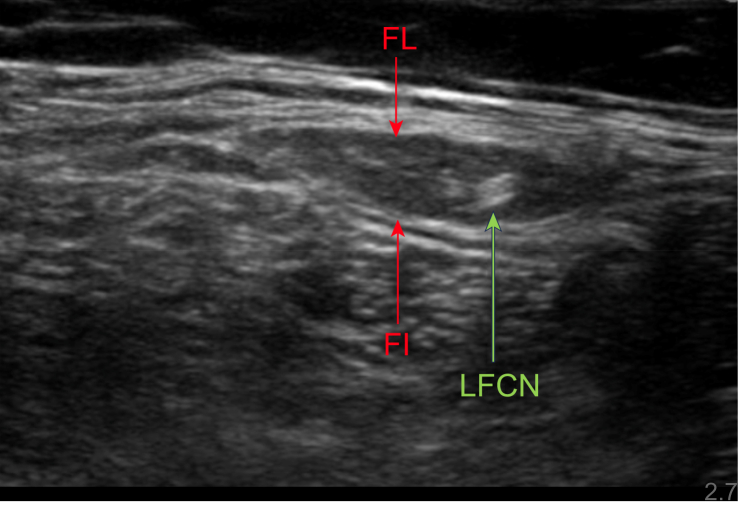
Ultrasound image showing the lateral femoral cutaneous nerve (LFCN) located between fascia lata (FL) and fascia iliaca (FI).

Ultrasound-guided infiltration is performed with the patient in the supine position using a high frequency transducer in the 7–14 MHz range. The lateral end is placed over the anterior superior iliac spine and the medial end is angled in a slight caudal direction over the inguinal ligament. The transducer is then moved in a mediocaudal direction to locate the LFCN. Once located, the transducer is moved medially to facilitate easier needle access. Under aseptic conditions, a 22-gauge NR fit needle is advanced in the longitudinal axis of the probe with the nerve in the short axis view. An LA and corticosteroid mixture is injected to achieve spread around the nerve and the mixture should resemble a doughnut as it surrounds the LFCN (Fig. 3, Fig. 1 online video).^28^ In our practice we use a combination of 1ml levobupivacaine 0.5% and triamcinolone 40 mg but other combinations such as methylprednisolone acetate and mepivacaine can be used. The volume of perineural injectate is usually limited to 2 ml.

#### Evidence for interventional techniques

The evidence to support the use of interventional techniques is mostly limited to observational studies and case reports that report positive effects (Table 3). A meta-analysis comparing LA and steroid ultrasound-guided infiltration with surgery found an 89% treatment success rate after infiltration of the LFCN with LA and corticosteroid. The authors reported no significant differences between surgery and infiltration, but infiltration was quicker, safe and less expensive than surgery.^29^ A systematic review found that symptoms associated with MP resolve or improve in 83% of patients after infiltration of the LFCN with LA and corticosteroid.^30^

**Table 3:** Interventional treatment options for meralgia paraesthetica. BSI, Bothersomeness Index; NRS, numerical rating scale; PIS, pain intensity score; PRF, pulsed radiofrequency lesioning; QOL, quality of life; VAS, visual analogue scale.

Intervention	Details of study	Outcome measures	Main results
Infiltration	Tagliafico and colleagues^28^ (2011) *N* = 20 Prospective observational study	Percentage technical success VAS (symptoms) VAS (QOL)	80% Technical success 20% Required repeat injection Significant reduction of VAS symptoms (*p*<0.001) from baseline of 8.1 (2.1) to 2.1 (0.5) at 2 months Significant reduction of VAS QOL (*p*<0.002) from baseline of 6.9 (3.2) to 2.3 (2.5) at 2 months
	Klauser and colleagues^31^ (2015) *N* = 20 Case series	VAS (symptoms)	Complete resolution: 75% of patients had a significant mean VAS decrease from 82 at baseline to 0 at 12 months (*p*<0.0001) Partial resolution: 25% of patients had a significant mean VAS decrease from 92 at baseline to 42 at 12 months (*p*<0.01)
Pulsed radiofrequency	Lee and colleagues^32^ (2016) *N* = 11 Case series	VAS (pain)	Significant reduction of VAS score from 6.4 (0.97) cm at baseline to 0.91 (0.70) cm, 0.82 (0.75) cm, 0.63 (0.90) cm and 1 (1.41) cm at 1, 3, 6 and 12 months after the pulsed radiofrequency procedure, respectively (*p*<0.001)
	Ghai and colleagues^33^ (2018) *N* = 5 Case series	NRS	Reduction of NRS from 8 (0.70) at baseline to 2.8 (1.3) post-procedure. Subsequent reduction to 2.2 (0.83), 1 (0.70), 0.75 (0.5), 0.5 (0.57), 0.33 (0.57), 0 (0) and 0 (0) at 3, 6, 9, 12, 15, 18, 25 months after PRF, respectively No calculation of significance
Radiofrequency ablation	Abd-Elsayed and colleagues^34^ (2020) *N* = 6 Case series	PIS	Significant % reduction (*p*<0.05) of average PIS to 75% post-procedure. Subsequent reduction to 60%, 58%, 51.4%, 40.50% and 37.5% at 1, 2, 3, 6 and 12 months post-procedure, respectively
Peripheral nerve stimulation	Langford and colleagues^35^ (2021) *N* = 1 Case report	PIS	Reduction in PIS from 8 to 0
	Dalal and colleagues^36^ (2021) *N* = 1 Case report	Percentage improvement in pain	2 Weeks after lead insertion: 90% 1 month after lead insertion: 100% 1 month after lead removal: 80%
Spinal cord stimulation	Barna and colleagues^37^ (2005) *N* = 1 Case report	Percentage satisfaction Percentage pain relief	Temporary device 100% satisfaction Significant reduction in pain Permanent device Nearly 100% pain relief
Surgical decompression	de Ruiter and colleagues^38^ (2012) Neurolysis (*n* = 10) Neurectomy (*n* = 8) Retrospective observational study	Percentage with complete pain relief Percentage satisfied with outcome Percentage bothered by numbness after neurectomy	Complete pain relief after: neurolysis: 60% neurectomy: 75% Satisfied with outcome: neurolysis: 100% neurectomy: 87.5% Bothered by numbness after neurectomy: no: 62.5% sometimes: 25% frequently:12.5%
	de Ruiter and colleagues^39^ (2015) Neurolysis (*n* = 8) Neurectomy (*n* = 14) Prospective observational study	Likert scale NRS BSI	Successful pain reduction (Likert 1 or 2) in: neurectomy: 93.3% neurolysis: 37.5% Significant difference between the two groups (*p*<0.0283) Neurolysis and neurectomy had non-significant reduction in NRS and BSI post-procedure (*p*=0.0683 and 0.0542, respectively)

### Neuromodulation

Electrical stimulation has a role in the management of chronic pain cases that have not been resolved with conservative measures. Various interventional studies have investigated the role of pulsed radiofrequency lesioning (PRF), radiofrequency ablation (RFA), peripheral nerve stimulation (PNS) and spinal cord stimulation in the management of MP (Table 3). Two retrospective case reviews of PRF have promising results.^32^^,^^33^ It has been postulated that electrode position and duration of PRF may influence the success of treatment. Large studies are required to investigate these parameters and the role of PRF in the treatment of MP.^33^

Abd-Elsayed and colleagues studied the effect of RFA using thermal denervation of the LFCN in six patients with MP.^34^ They reported a 40.5% reduction in the pain intensity score 6 months post-procedure with no associated complications.^34^

The evidence for the use of PNS as a treatment for MP is limited to case reports using a temporary externalised system. One study showed an improvement of the NRS from 8/10 to 2/10 30 days after PNS lead removal^40^ whilst another study reported an 80% improvement in pain 1 month after lead removal.^36^ Other authors postulated that the PNS system modulates central sensitisation, allowing for relief of symptoms after PNS removal, preventing the need for implanting a permanent system.^37^

Spinal cord stimulation is an invasive surgical procedure that has been used for the treatment of MP where conservative treatments have failed. Barna and colleagues reported a case in which a patient had 100% pain relief, significant functional improvement and no adverse effects 8 months after implantation with a permanent spinal cord stimulator.^37^ Although the role of electrical stimulation in managing MP is promising, there is a lack of published evidence and its role in the management of MP remains unclear.

### Surgical

Meralgia paraesthetica is treated surgically when conservative measures fail. There are two main surgical procedures: neurolysis and neurectomy. Neurolysis is the decompression of the LFCN at the level of the inguinal ligament and neurectomy is the transection of the LFCN.

de Ruiter and colleagues published two studies comparing the outcomes after neurolysis and neurectomy. Both studies found that neurolysis and neurectomy were effective in the treatment of MP, with neurectomy producing a higher success rate.^38^^,^^39^ Although neurolysis is usually carried out as the first-line surgical option to avoid the numbness and deafferentation pain associated with neurectomy, a retrospective review by de Ruiter and colleagues found no reports of numbness or deafferentation pain in 10 patients undergoing neurectomy.^38^ Khalil and colleagues reported similar success with decompression and neurectomy.^30^

## Conclusions

There is often a delay in diagnosis of MP because of its variable presentation and lack of knowledge about the condition. Diagnosis is usually clinical but ultrasound, SSEP, SNAP and SNCV may be helpful in its diagnosis. Most patients will respond to conservative management. Ultrasound-guided injection of the LFCN with LA and corticosteroid can be considered as an adjunct to conservative measures. More invasive measures have been used when ultrasound injection fails although larger scale studies are required before these can be recommended as suitable treatments.

## MCQs

The associated MCQs (to support CME/CPD activity) will be accessible at www.bjaed.org/cme/home by subscribers to *BJA Education*.

## Declaration of interests

The authors declare that they have no conflicts of interest.
